# Anti-Gastric Cancer Activity of the Cell-free Culture Supernatant of Serofluid Dish and *Lactiplantibacillus plantarum* YT013

**DOI:** 10.3389/fbioe.2022.898240

**Published:** 2022-05-23

**Authors:** Rentao Zhang, Zhongkun Zhou, Yunhao Ma, Kangjia Du, Mengze Sun, Hao Zhang, Hongyuan Tu, Xinrong Jiang, Juan Lu, Lixue Tu, Yuqing Niu, Peng Chen

**Affiliations:** School of Pharmacy, Lanzhou University, Lanzhou, China

**Keywords:** AGS cells, apoptosis, cytotoxic effect, *lactiplantibacillus plantarum*, serofluid dish

## Abstract

Cancer is second only to heart disease as a cause of death, despite improvements in its early diagnosis and precision medicine. Due to the limitations of commonly used anticancer methods such as surgery, radiotherapy and chemotherapy, biological therapy, especially probiotics such as lactic acid bacteria, has received widespread attention. *Lactobacillus* has been proven to inhibit the proliferation of a variety of cancer cells. In this work, the effects of the cell-free culture supernatant of serofluid dish (CCS1) and the cell-free culture supernatant of *Lactiplantibacillus plantarum* YT013 (CCS2) isolated from serofluid dish on AGS, HCT116, HepG2 and PANC-1 cells were investigated. Based on the CCK-8 assay, CCS1 and CCS2 were shown to suppress the growth of cancer cells in a concentration-dependent manner. The IC_50_ values of CCS2 of AGS, HCT116, HepG2 and PANC-1 cells were 346.51 ± 35.28, 1207.69 ± 333.18, 650.94 ± 123.78 and 808.96 ± 126.27 μg/ml, respectively. In addition, the results of fluorescence microscopy showed that CCS2 changed cell morphology and treated with CCS2 (200, 400 and 800 μg/ml) for 48 h, AGS cell apoptosis was quantitatively surveyed by flow cytometry, showing 25.0, 34.1, and 42.6% total apoptotic cells. Moreover, western blotting confirmed that BAX, BAD and Caspase-3/8/9 were significantly upregulated and that BCL-2 was significantly downregulated in AGS cells treated with CCS2. These results indicated that CCS2 might lead to apoptosis via the endogenous mitochondrial apoptotic pathway. In summary, *Lactiplantibacillus plantarum* YT013 may be considered a good candidate for anticancer therapies.

## Graphical Abstract

The overall mechanisms for the induction of apoptosis in AGS cells by the CCS2 prepared from the selected *L. plantarum* YT013 were summarized as the picture.
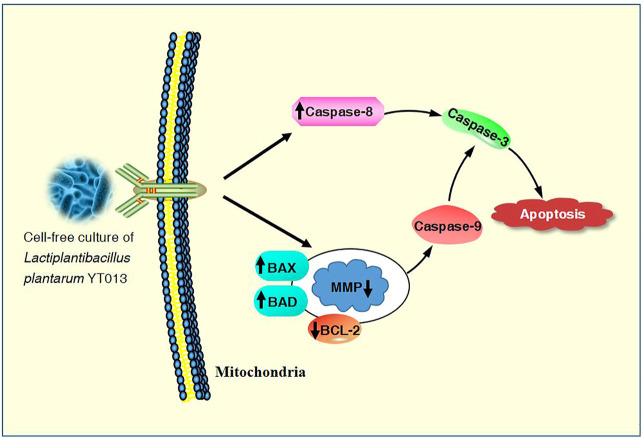

## Introduction

Gastric cancer is a common disease threatening human health (de Boer et al., 2018). According to GLOBOCAN 2020 compiled by the International Agency for Research on Cancer (IARC), gastric cancer is the fourth leading cause of death and the fifth leading cause of morbidity among cancers worldwide (Ferlay et al., 2018; Sung et al., 2021). The predominant gastric cancer diagnosed cases are a type referred to as adenocarcinoma of the stomach and develop from the cells of superficial stomach mucous membrane (Wang et al., 2019). In recent years, the age of onset of gastric cancer has decreased, and the morbidity has increased (Jian et al., 2020). As most gastric cancer patients are diagnosed at the late stage, the response to chemotherapy is often very poor, resulting in long-term and serious side effects and a very low 5-years survival rate. It is gratifying that compared with traditional chemotherapy drugs, natural product drugs are widely considered because of their safety, effectiveness, stable effects and fewer side effects.

Probiotics have been widely used as a substitute for traditional drugs to maintain intestinal homeostasis and prevent diseases. As probiotics, *Lactobacillus* has been proven to have good anticancer effects (Reid 2016). Previous investigations have revealed that the presence of *Lactobacillus* in the gastrointestinal tract can improve the composition of intestinal flora, promote peristalsis and reduce the residence time of carcinogens (Kim et al., 2019). In addition, recent studies have confirmed that lactobacilli have a strong and lasting cytotoxic effect on cervical cancer cells, which can inhibit their migration by upregulating the protein of E-cadherin (Li et al., 2017). *Lactobacillus* can also inhibit the proliferation, migrate and promote apoptosis of colorectal cancer cells (Eslami et al., 2019; Lin et al., 2019) and compete with *Helicobacter pylori* (*H. pylori*) in the mucous layer of the human stomach, thus inhibiting its colonization (Yang et al., 2021). Moreover, *Lactobacillus* extracts induced apoptosis and prevented cell cycle progression in S-phase, showing that they had anti-proliferative activity on HT-29 and LT-97 cell lines. In another study, some strains of *Lactobacillus* could mitigate *H. pylori* infection by preventing its adhesion to epithelial cells and producing some metabolites (Fang et al., 2019; Song et al., 2019). One of the common probiotics in the gastrointestinal tract *Lacticaseibacillus gasseri* could ameliorate *H. pylori*-induced inflammation by decreasing the expression of *BCL-2*, *β-catenin*, *integrin α5*, and *integrin β1* genes in AGS cells (Yarmohammadi et al., 2021). These investigations indicated that probiotics extraordinary increases in bioavailability and is efficient, ecologically safe and low cost for anticancer therapies.

Serofluid dish (*Jiangshui* in Chinese) is a kind of traditional fermented vegetable food in Northwest China with a long history, and contains many microorganisms, mainly including *Lactobacillus* and *Acetobacter* (Chen et al., 2016; Liu et al., 2019; Zhou et al., 2021). According to our previous studies, the cell-free culture supernatant of serofluid dish and *Lactobacillus* had good inhibitory effects on the proliferation of colorectal cancer cells. Additionally, compared with traditional chemotherapy drugs, *Lactobacillus*, as a natural anticancer resource, have the advantages of safety, high efficiency and fewer side effects, and may play an extraordinarily important role in the prevention and treatment of gastric cancer. *Lacticaseibacillus casei* LH23, isolated from traditional fermented food, plays an anticancer role by reversing a variety of biological characteristics of cervical cancer cells, such as cell proliferation, apoptosis and migration (Hu et al., 2021). It has also been shown that the exopolysaccharides (EPS) of *Lactiplantibacillus plantarum* (*L. plantarum*) in naturally fermented foods can effectively reduce insulin resistance in HepG2 cells, *a*-amylase activity and upregulate the expression of *GLUT-4*, *Akt-2* and *AMPK* genes (Huang et al., 2020). Currently, little is known about the role of serofluid dish in the prevention and treatment of gastric cancer, and further study is urgently needed. This research was performed to assess the anticancer effect of the cell-free culture supernatant of serofluid dish and *L. plantarum* YT013 on gastric cancer AGS cells and to reveal the potential mechanism involved.

## Methods and Materials

### Preparation of the Cell-free Culture Supernatant of Serofluid Dish (CCS1)

The fermented serofluid dish was used as the experimental raw material and transferred to the laboratory in a low-temperature incubator. The preparation method of experimental material was referred to the research of Riaz Rajoka et al. (Riaz Rajoka et al., 2019). Solids were removed, and the material was centrifuged (2,000 ×g, 20 min, 4°C). The pellet was added to de Man, Rogosa and Sharpe (MRS) broth (Solarbio Science & Technology, Beijing, China) at an equal volume and cultured at 37°C for 24 h. The solution was centrifuged (2,000 ×g, 20 min, 4°C), after which the supernatant was discarded. The pellet was resuspended in sterile phosphate-buffered saline (PBS) and centrifuged (2,000 ×*g*, 20 min, 4°C). Roswell Park Memorial Institute (RPMI) 1640 medium or Dulbecco’s Nodified Eagle Medium (DMEM) (Solarbio, Beijing, China) was added to adjust the bacterial concentration to 10^9^ CFU/ml, and the sample was cultured at 37°C, 120 rpm for 12 h. The number of cells (CFU/ml) was determined by serial dilution and coated plate on MRS agar. Before use, the pH value of the material was adjusted to 7.2 with 1.0 mol/L NaOH, and then centrifuged (2,000 ×*g*, 20 min, 4°C) and passed through 0.22-μm filters twice.

### Preparation of the Cell-free Culture Supernatant of *L. plantarum* YT013 (CCS2)

The lactic acid bacteria strain *L. plantarum* YT013 was preserved at China Center for Type Culture Collection (CCTCC No. M2018775). The fermentation supernatant of *L. plantarum* YT013 was used as the experimental material, and the preparation method was referred to the research of Riaz Rajoka et al., with a brief modification (Riaz Rajoka et al., 2019). First, the supernatant of *L. plantarum* YT013 was collected by centrifugation (2,000 ×*g*, 15 min, 4°C), and the number of bacteria was calculated based on UV spectrophotometry (Yoke Instrument Co., Ltd., Shanghai, China). A specific number of bacteria cells were added to RPMI 1640 or DMEM medium and cultured at 37°C for 24 h. And then the supernatant of *L. plantarum* YT013 was centrifuged (2,000 ×*g*, 15 min, 4°C). The pH value of the supernatant obtained after centrifugation was adjusted to 7.2 with 1.0 mol/L NaOH. Added protease (Solarbio, Beijing, China) to the supernatant at a final concentration of 1.0 mg/ml and then incubated the mixture at 37°C for 30 min to remove all proteins. Then, the protease was inactivated by heating, and the sample was centrifuged (2,000 ×*g*, 15 min, 4°C) and freeze-dried to obtain CCS2. The CCS2 solution was filtered through 0.22-μm filters twice before experiments.

### Cell Lines and Growth Condition

AGS, HCT116, HepG2 and PANC-1 cell lines were purchased from American Type Culture Collection (ATCC Lot: 70012225, 70019042, 70015966, 70018880). HL-7702 and GES-1 cell lines were supplied by the School of Pharmacy, Lanzhou University. AGS, HCT116, HepG2 and HL-7702 cells were cultured in RPMI 1640 medium. PANC-1 and GES-1 cells were cultured in DMEM medium. All media included 10% fetal bovine serum (Sijiqing Biologic Co. Ltd., Zhejiang, China) and 1% penicillin/streptomycin (Solarbio, Beijing, China), and incubation was carried out at 37°C and 5% CO_2_.

### Cells Viability Assay

The anti-proliferation activities of CCS1 and CCS2 on cancer cells were detected using the CCK-8 kit (Coolaber Technology Co., Ltd., Beijing, China) assay (Shao et al., 2017). Cells were inoculated into 96-well plates and cultured overnight, treated with 100 μL of CCS1 (10, 20, 30, 40 and 50% v/v) or CCS2 (200, 400, 600, 800 and 1000 μg/ml) and then cultured for 48 h at 37°C with 5% CO_2_. The medium alone was used as a control, and each well was performed in triplicate. At the end of treatment, CCK-8 working liquid (10 μL/well) was added and incubated for another 2 h, and the absorbance values of the wells were measured at 450 nm using a microplate reader (Perlong New Technology Co., Ltd.,Beijing, China). $\mathrm{Inhibitory}\;\mathrm{rate}\left(\%\right)=\left[\mathrm{1-}\frac{A_{\mathrm{sample}}-A_{\mathrm{blank}}}{A_{\mathrm{control}}-A_{\mathrm{blank}}}\right]\mathrm{\times 100,}$ where $A_{\mathrm{control}}$ and $A_{\mathrm{blank}}$ are the absorbance values without CCS2 or cells, respectively. In addition, the half maximal inhibitory concentration (IC_50_) value of CCS2 on the four cell lines after 48 h was calculated.

### Migration Assay

Observation of changes in nuclear morphology of AGS cells after CCS2 treatment by Hoechst 33258 staining (Xiao et al., 2020). Cancer cells were cultured in 24-well plates at 80,000 cells per well and treated with CCS2 (0, 200, 400, and 800 μg/ml) for 48 h. The cells were treated with 4% paraformaldehyde fixative for 15 min; the fixative was removed with PBS. An appropriate volume of Hoechst 33258 (Solarbio, Beijing, China) working liquid was added, and the mixture was placed in a dark room for 10 min, followed by washing three times with PBS. The stained cells were analyzed with a fluorescence microscope (Carl Zeiss AG, Oberkochen, Germany).

### Mitochondrial Membrane Potential Analysis

Once the mitochondrial membrane potential (MMP) is lost, cells will enter an irreversible process of apoptosis (Lin et al., 2017). AGS cells were cultured in 24-well plates with CCS2 (0, 200, 400, and 800 μg/ml) for 48 h and washed three times with PBS. Then, an appropriate volume of JC-10 (Solarbio, Beijing, China) working liquid was added to cover all cells and incubated at 37°C in 5% CO_2_ for 15 min. The supernatant was removed, and the cells were washed three times with washing solution and analyzed by fluorescence microscopy.

### Flow Cytometry Analysis

The apoptosis was detected by fluorescent cell counts using the Annexin V Alexa Fluor 488/PI kit (Solarbio, Beijing, China) following the manufacturer’s instruction (Du et al., 2022). Briefly, the AGS cells were cultured in 6-well culture plates at 8×10^5^ cells/well and treated with CCS2 (0, 200, 400 and 800 μg/ml) for 48 h. After incubation, the cells were treated with trypsin without EDTA and washed twice with precooled PBS. The cells were treated by the Annexin V Alexa Fluor 488/PI kit. Then the flow cytometry (FACSCalibur, BD Biosciences, Mountain View, CA, United States) was used to analyze the samples to detect Annexin V- and PI-positive subsets. Further analysis was performed with Flow Jo V10 software.

### Western Blotting Assay

The Western blotting assay was performed as described by Sun et al. (Sun et al., 2021). AGS cells were cultured in 6-well plates at 8×10^5^ cells/well, and the cells were treated with CCS2 (400 μg/ml) for 48 h at 37°C with 5% CO_2_. Total protein was extracted from AGS cells with RIPA lysis buffer (Solarbio, Beijing, China) containing 1% PMSF and protein phosphatase inhibitor (Solarbio, Beijing, China). A BCA protein detection kit (Coolaber, Beijing, China) was used to determine the protein concentration. After 12% sodium dodecyl sulfate-polyacrylamide gel electrophoresis (SDS–PAGE), proteins were transferred to polyvinylidene fluoride (PVDF) membrane, which was blocked for 2 h in 1× TBST containing 0.1% Tween 20 and 5% skim milk. GAPDH was used as an internal reference. Antibodies (Bioss, Beijing, China) of BAD, BAX, BCL-2, Caspase-3, Caspase-8 and Caspase-9 were applied, and the membrane was then incubated at room temperature for 2 h with an HRP-labeled secondary antibody, after cleaned with TBST, followed by autoradiography, imaging and recording. The images were analyzed with ImageJ software.

### Statistical Analysis

All experiments were performed in duplicate at a minimum, and the average values were reported, and the data were analyzed by SPSS 22.0. Significant differences were determined using the *t*-test. Statistical significance is expressed as *p < 0.05, **p < 0.01, and ***p < 0.001 vs. the control groups.

## Results

### CCS1 and CCS2 Display Potent Anti-proliferative Activity in the AGS, HCT116, HepG2 and PANC-1 Cell Lines

**FIGURE 1:**
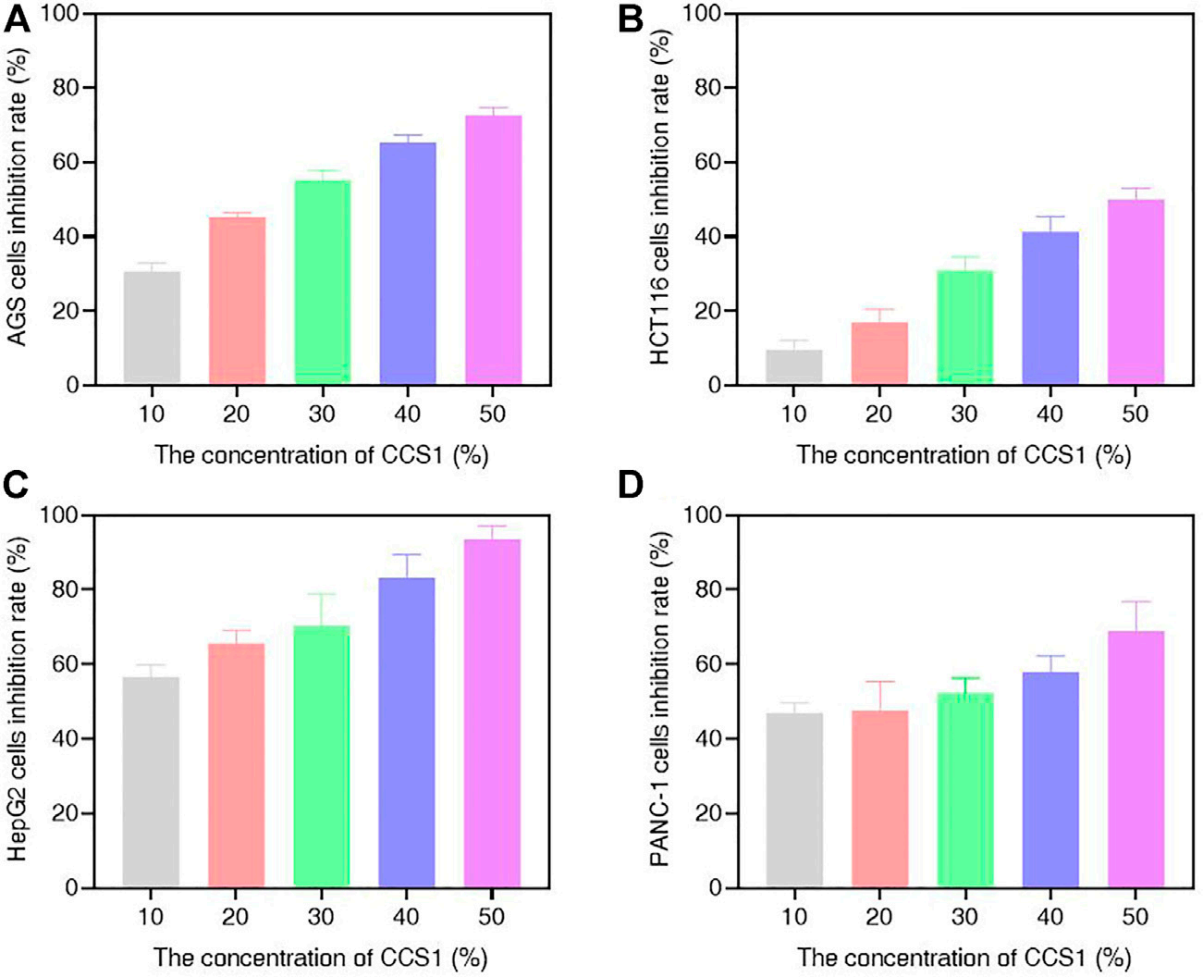
Suppressive effect of CCS1 on the proliferation of four kinds of digestive tract digestive tract cancer cells. The results were obtained after treatment with CCS1 for 48 h **(A–D)** CCK-8 results for AGS, HCT116, HepG2 and PANC-1 cells. Data are expressed as the average of three independent experiments.

**FIGURE 2:**
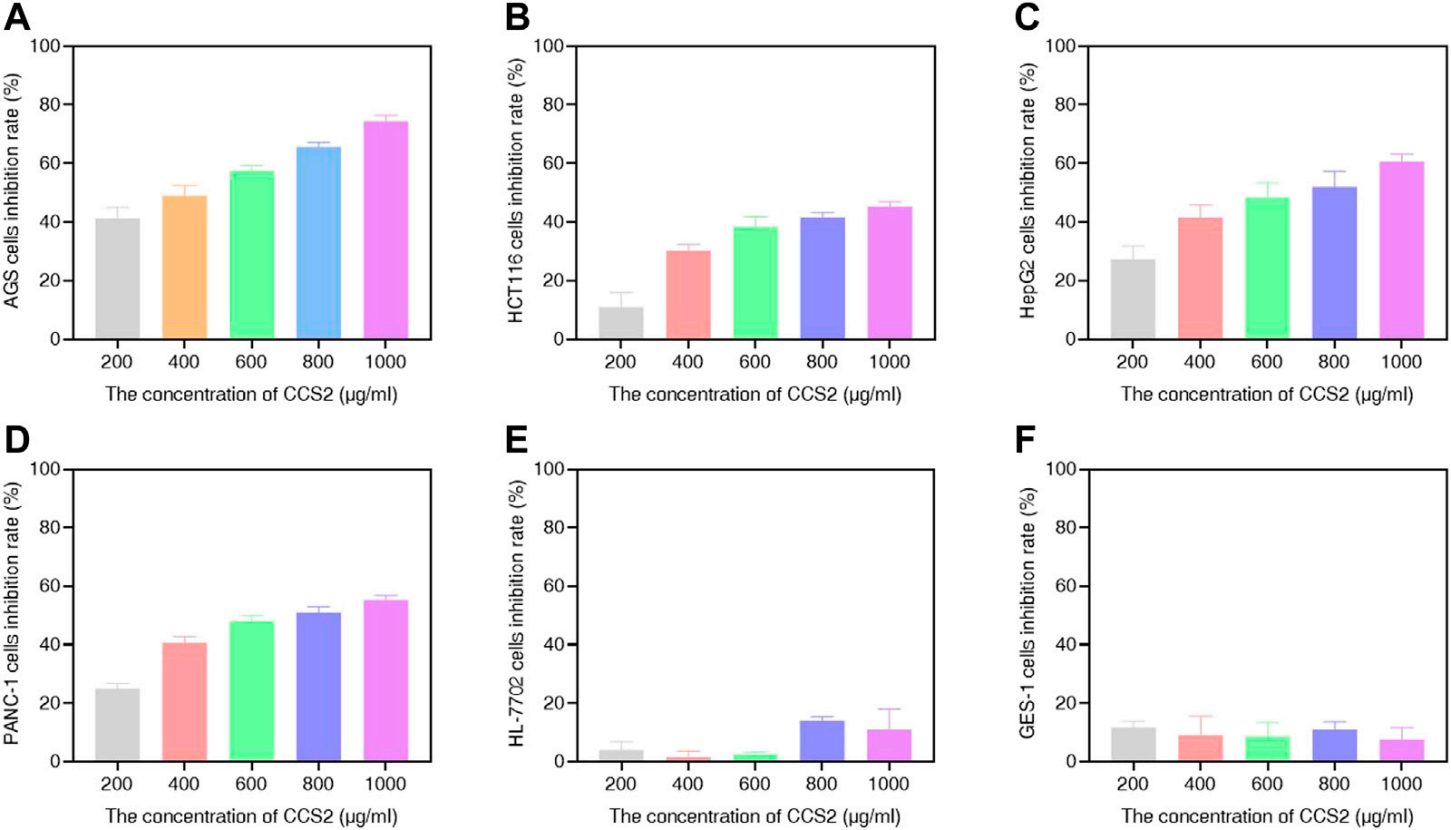
Suppressive effect of CCS2 on the proliferation of four kinds of digestive tract digestive tract cancer cells. The results were obtained after treatment with CCS2 for 48 h **(A–F)** CCK-8 results for AGS, HCT116, HepG2, PANC-1, HL-7702 and GES-1 cells. Data are expressed as the average of three independent experiments.

In this study, different concentrations of CCS1 and CCS2 were evaluated for anti-proliferative activity against four kinds of digestive tract cancer cells (AGS, HCT116, HepG2 and PANC-1) firstly, and results showed that both had inhibitory effect on the growth of the four types of cancer cells (Figure 1 and Figures 2A–D). At the same concentration, CCS2 had the highest inhibition rate on AGS cells, followed by HepG2 and PANC-1. The IC_50_ values of AGS, HCT116, HepG2 and PANC-1 cells were 346.51 ± 35.28, 1207.69 ± 333.18, 650.94 ± 123.78 and 808.96 ± 126.27 μg/ml, respectively. Therefore, AGS cells were selected for further study. Moreover, the toxicity of CCS2 was evaluated on liver cell line HL-7702 and gastric mucosa cell line GES-1 (Figures 2E,F). The inhibition rates of HL-7702 and GES-1 were lower than 20% even when the concentration of CCS2 reached 1000 μg/ml, indicating CCS2 had selective anti-proliferative activity.

### CCS2 Induced Morphological Changes in the AGS, HCT116, HepG2 and PANC-1 Cell Lines

**FIGURE 3:**
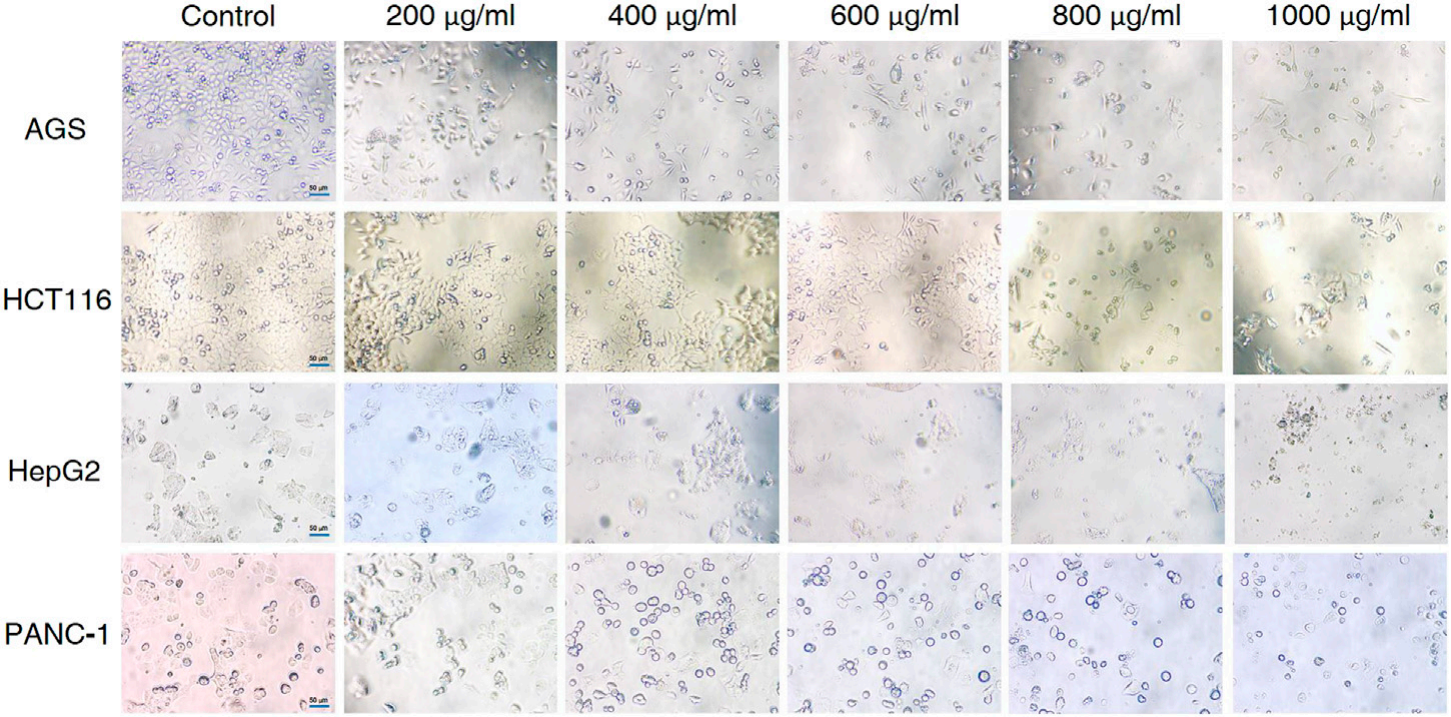
Effect of CCS2 on the morphology of four kinds of digestive tract cancer cells. Cell photographs of four kinds of digestive tract cancer cells treated with CCS2 (0, 200, 400, 600, 800, and 1000 μg/ml) for 48 h. Scale bar = 50 μm.

The effects of different doses of CCS2 on the morphology of cancer cell lines were observed (Figure 3). The control groups grew well on the surface of the cell culture dish, whereas the sizes of cancer cells decreased and the cells became rounded, contracted, loosely arranged and poorly adhered with increased doses of CCS2. Eventually, most of the cancer cells died under high concentrations.

### CCS2 Induced Apoptosis of AGS Cells by Hoechst 33258 Staining Analysis

**FIGURE 4:**
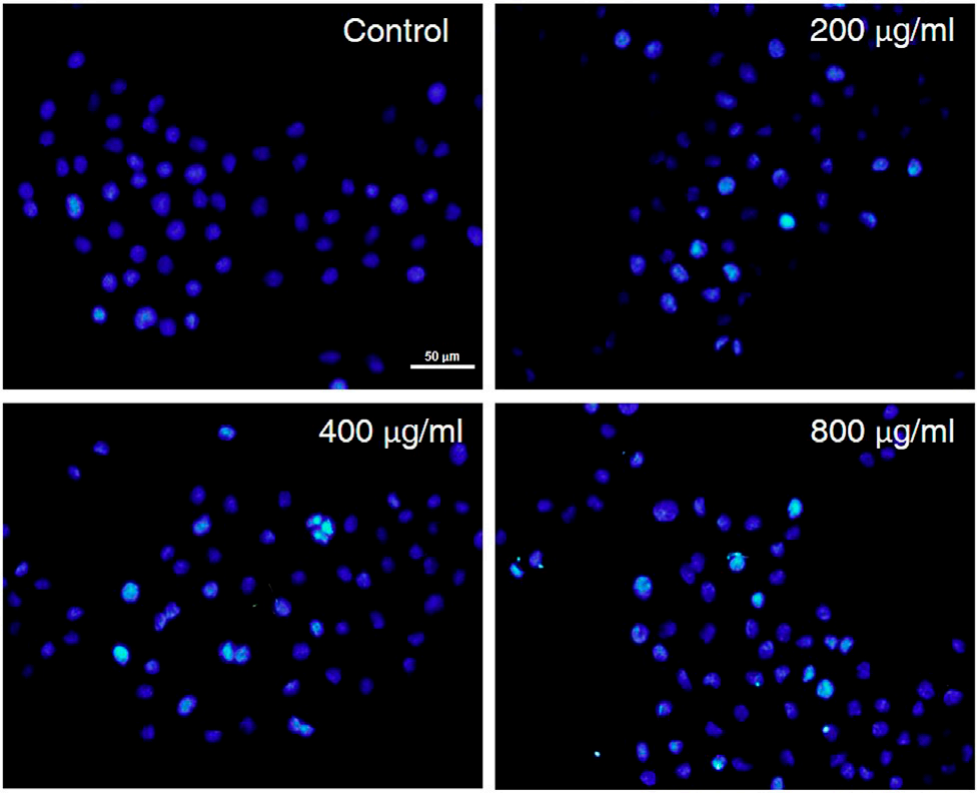
The influence of CCS2 on AGS cell morphology. AGS cells were treated with fluorescent dyes and observed under fluorescence microscopy after treatment without or with CCS2 (0, 200, 400 and 800 μg/ml) for 48 h. Scale bar = 50 μm.

The treated AGS cells were stained and observed by a fluorescence microscope to evaluate the cytotoxicity and morphological changes of the cells (Riaz Rajoka et al., 2018). To further understand whether the anti-proliferative activity of CCS2 on AGS cells was due to apoptosis induction, AGS cells were treated with CCS2 at different concentrations for 48 h and the apoptosis was visualized after Hoechst 33258 staining. As is shown in Figure 4, untreated cells showed that the nuclei were similar in size, and uniformly and lightly stained. Nevertheless, AGS cells treated with CCS2 at different concentrations showed typical morphological changes (Ma et al., 2022), such as chromatin condensation, densely stained nuclei, and apoptotic bodies. These results suggested that apoptosis could be the potent mechanism of anti-proliferative activity.

### CCS2 Caused Mitochondrial Membrane Potential Changes in AGS Cells

**FIGURE 5:**
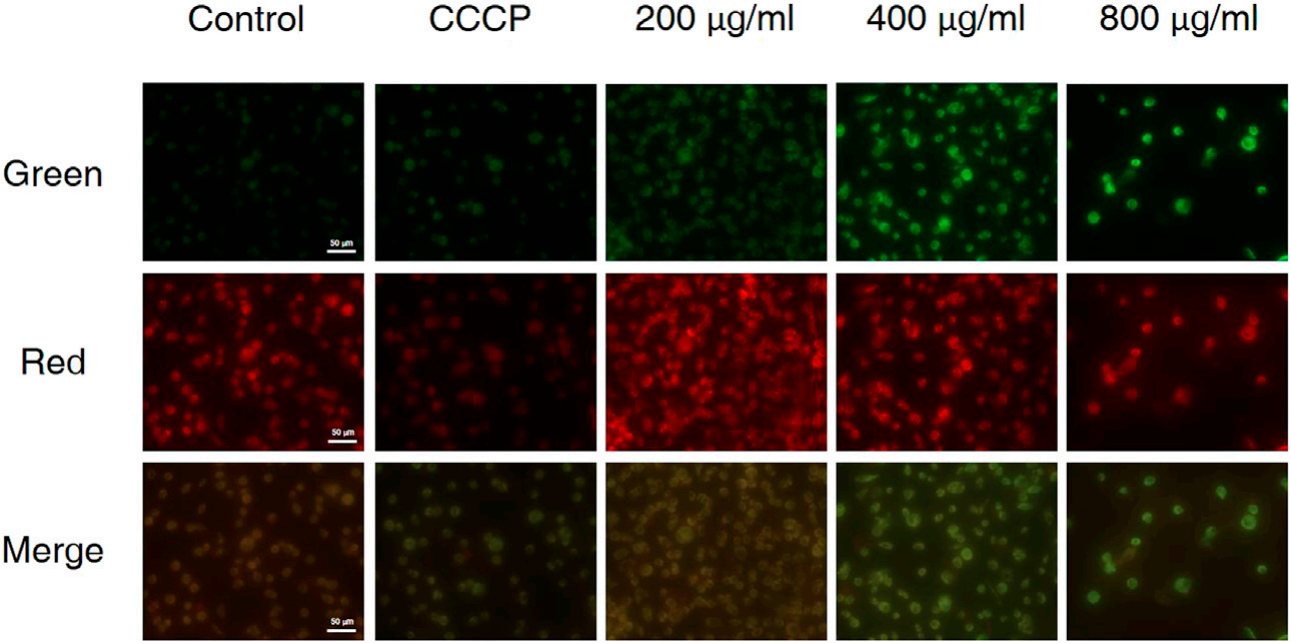
Treatment of AGS cells with CCS2 induced disruption of mitochondrial membrane potential. AGS cells were treated with different doses of CCS2 (0, 200, 400 and 800 μg/ml) for 48 h. Scale bar = 50 μm.

The decrease in mitochondrial transmembrane potential indicates that if apoptosis was induced via the mitochondrial pathway and the integrity of the mitochondrial membrane will be disrupted at the early stage of apoptosis (Riaz Rajoka et al., 2019). In living cells, MMP is high, and JC-10 accumulates in the matrix of mitochondria to form a polymer, resulting in red fluorescence; when MMP is low, JC-10 cannot aggregate in the matrix, remains a monomer and produces green fluorescence. As is shown in Figure 5, carbonyl cyanide m-chlorophenyl hydrazone (CCCP) was used as the positive control, when AGS cells were treated with different concentrations of CCS2, the MMP of AGS cells decreased significantly in a dose-dependent manner. These results showed that CCS2-induced apoptosis was associated with mitochondrial-mediated pathways.

### CCS2-Induced Apoptosis of AGS Cells by Flow Cytometry Analysis

**FIGURE 6:**
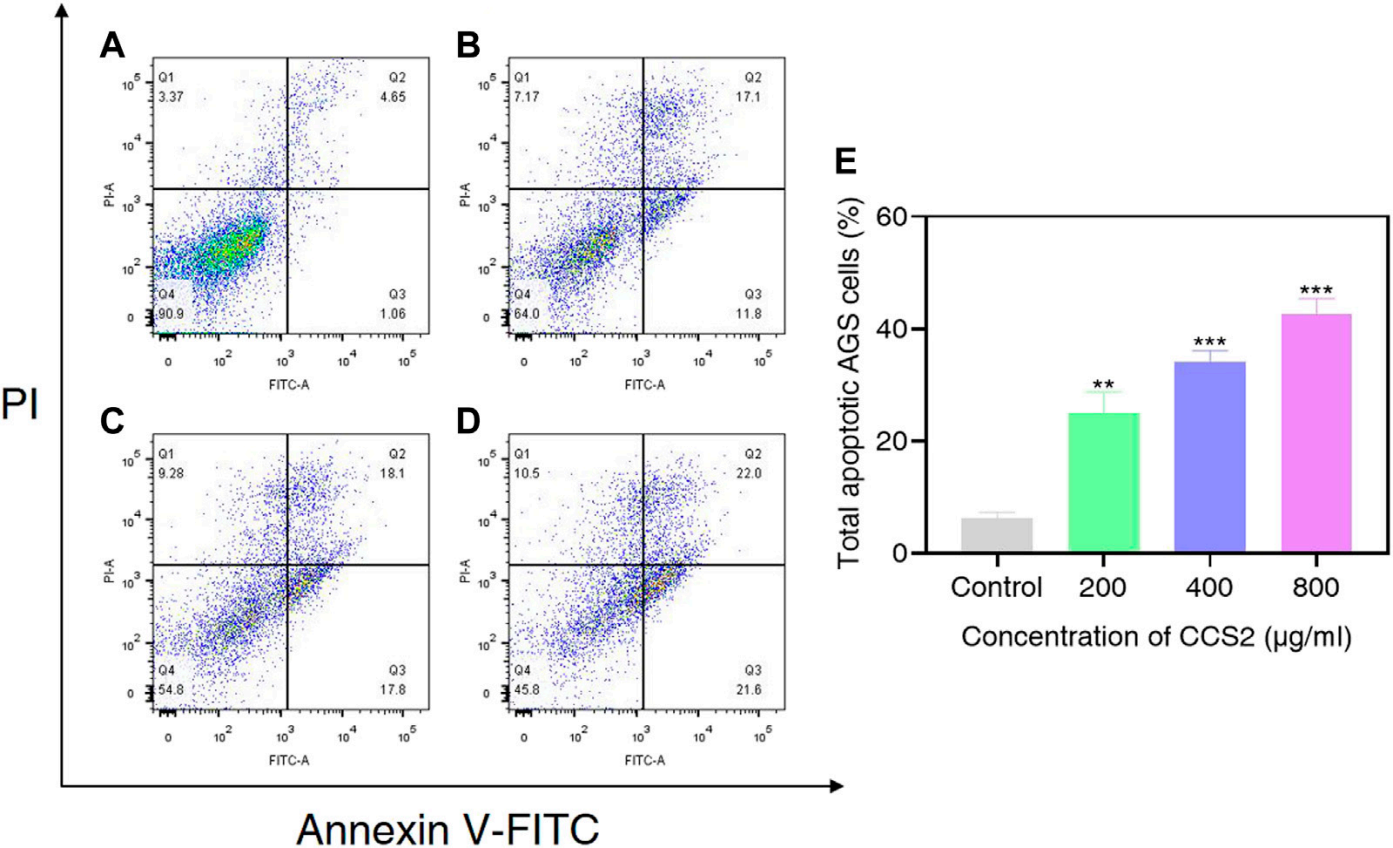
AGS cells were analyzed by flow cytometry. **(A–D)** Photographs of AGS cells without or with CCS2 (200, 400 and 800 μg/ml) treatment for 48 h. **(E)** The percentage of total apoptotic cells. Data are expressed as the average of three independent experiments. *p < 0.05, **p < 0.01, ***p < 0.001 compared with control.

The accurate modes of cell death could be characterized by flow cytometry analysis (Riaz Rajoka et al., 2019). To further evaluate cell death signal quantitatively, CCS2 treated AGS cells were analyzed by flow cytometry. As shown in Figure 6, the proportion of apoptotic cells increased from 6.3% (control) to 25.0% (200 μg/ml CCS2), 34.1% (400 μg/ml CCS2) and 42.6% (800 μg/ml CCS2), respectively. Furthermore, the proportion of necrotic cells increased after CCS2 treatment. Taking the relatively higher percentage of late apoptosis into consideration, it was speculated that the necrosis of cells after treatment might be related to the fragmentation of DNA. Thus, apoptosis may be the main cause of cell death instead of necrosis.

### CCS2 Regulated the Expression of Proteins in the Caspase-dependent Endogenous Mitochondrial Apoptosis Pathways

**FIGURE 7:**
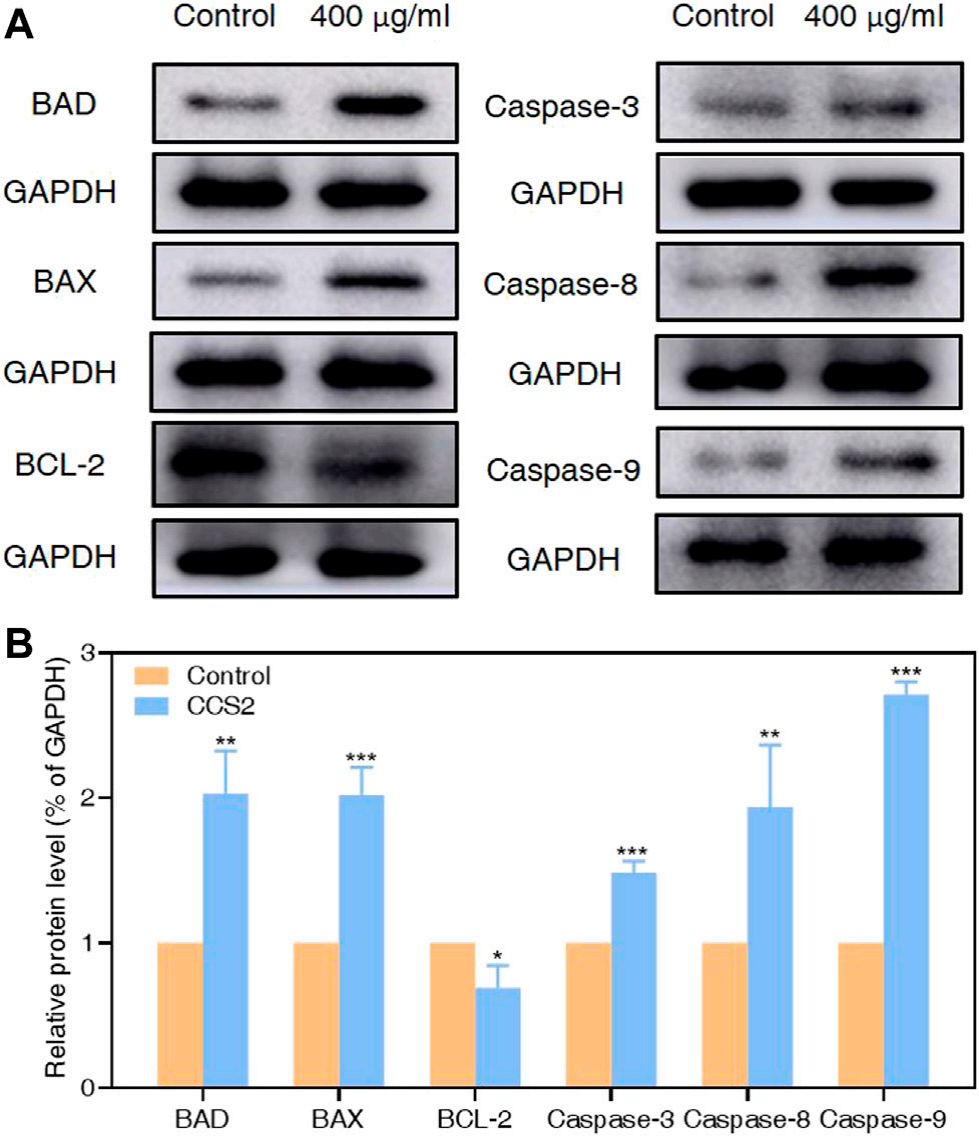
Relative protein expression of apoptosis-related proteins in AGS cells by western blotting analysis. Through western blotting analysis, the effect of CCS2 treatment on the protein expression of BAD, BAX, BCL-2, Caspase-3, Caspase-8 and Caspase-9 was determined. **(A)** Expression of apoptosis-related proteins was determined. **(B)** The histogram represents relative protein levels normalized to GAPDH for BAD, BAX, BCL-2, Caspase-3, Caspase-8 and Caspase-9. Data are expressed as the average of three independent experiments. Data are expressed as the average of three independent experiments. *p < 0.05, **p < 0.01, ***p < 0.001 compared with control.

It has been proved that the Caspase family plays a central role in the major execution of various apoptotic responses (Xiao et al., 2020). Next, western blot experiments were used to verify the effect of CCS2 on the apoptosis-related pathways. As shown in Figure 7, the protein expression levels of BAX, BAD, Caspase-3, Caspase-8 and Caspase-9 increased significantly while BCL-2 decreased after treatment with CCS2 for 48 h. These results indicated that the apoptosis of AGS cells induced by CCS2 was mediated by the internal mitochondrial apoptosis pathway.

## Discussion

Cancer is an important cause of death, and has brought huge burdens to countries around the world (Bray et al., 2018). Cancer treatment involves many different molecular mechanisms. Although many *in vitro* and *in vivo* studies have been carried out on the anticancer effects of probiotics, knowledge of their exact molecular mechanisms is incomplete (Saber et al., 2017). In our research, we assessed the influence of CCS1 and CCS2 on the growth of AGS, HCT116, HepG2 and PANC-1 cell lines and investigated their anti-proliferation activities in a concentration-dependent manner (Figures 1, 2). The results indicated that CCS2 could inhibit the proliferation activities of the four cell lines without affecting normal cells.

The treated cancer cells were stained and observed by fluorescence microscope is an appropriate way to assess morphological changes and cytotoxicity of cell chromatin and membranes (Haghshenas et al., 2015). To assess the changes of CCS2-induced on the morphology of AGS cells, a Hoechst 33258 assay was conducted. After cells were treated with CCS2, the chromatin was dense, deeply stained, and half-moon- or cap-shaped (bright staining) (Figure 4), and the structure formed by apoptotic bodies were also noted; these are characteristics of apoptotic cells (Galluzzi et al., 2018). Apoptosis and its related signaling pathways are important reasons for the strong rate of cell loss in cancer, with a strong impact on the development of cancer. Additionally, apoptosis can be used as a defense mechanism for cancer progression (Haghshenas et al., 2015; Jorgensen et al., 2017). Mitochondria are recognized to be the bioenergetic and metabolic center essential to life (Chen et al., 2014; Elumalai et al., 2012), and the decrease in MMP is an early manifestation of apoptosis. Cells will enter an irreversible process of apoptosis, as the MMP is lost (Bertheloot et al., 2021). For cells stained with JC-10, a change from red to green occurs with high to low MMP (Chen et al., 2014). Stained results showed that compared with the control groups, the cells treated with CCS2 indicated stronger green and weaker red fluorescence (Figure 5), which correlated positively with the concentration. However, it is not entirely convincing to judge apoptosis only by the experimental results obtained by fluorescence microscopy. Therefore, the accurate pattern of cell death was confirmed by flow cytometry (Haghshenas et al., 2015). After double staining, the total apoptosis rate (6.3, 25.0, 34.1, and 42.6%, respectively) of cells treated with different concentrations of CCS2 were detected by flow cytometry. Flow cytometry with Annexin V–PI staining was used to confirm that CCS2 caused apoptosis in a concentration-dependent manner (Figure 6). Studies have proven that the cell-free supernatant of *Lacticaseibacillus brevis* prevents proliferation and promotes apoptosis in breast cancer (Malik et al., 2018). In another study, found that *Lacticaseibacillus casei* SR1, SR2, and *Lacticaseibacillus paracasei* SR4 act against cervical cancer cells via the intrinsic mitochondrial apoptotic pathway inducing apoptosis (Riaz Rajoka et al., 2018). Apoptosis refers to the phenomenon of cell suicide that is closely regulated; it is a process of cell-autonomous death controlled by genes and an important mechanism to maintain the internal environment (Mohammad et al., 2015). This is a milestone in cancer treatment because cancer cells can be controlled by inducing apoptosis (Evan and Vousden, 2001). BCL-2 family proteins are a pivotal influencing factor for cancer cell mitochondria-mediated apoptosis. By regulating the production of cytochrome c and activating Caspase, the cellular response to death signals can be enhanced, inducing apoptosis (Chen et al., 2015; Sungur et al., 2017). Caspase is a cysteine aspartate-specific protease present in the cytoplasm that is specifically sensitized in the progression of apoptosis (Fan et al., 2005). Caspase-3 is the most commonly studied apoptotic protein, and Caspase-9 is necessary to activate Caspase-3 (Nuñez et al., 1998). Caspase-3 is a responder that further promotes the apoptosis signal after activation by a promoter (Van Opdenbosch and Lamkanfi, 2019). Among them, Caspase-8 and Caspase-9 are called promoter Caspases, which are related to cell signal transmission and apoptosis induction (Shafie et al., 2013). Western blotting results showed that treated with CCS2, protein expression levels of BAX and BAD were upregulated, BCL-2 was downregulated, and Caspases-3/8/9 were upregulated. Previous studies showed that *Lacticaseibacillus rhamnosus* strains acquired from human breast milk inhibit the proliferation and promote apoptosis in HeLa cells by upregulating BAD, BAX, and Caspase-3/8/9 and downregulating BCL-2 (Riaz Rajoka et al., 2019). These results reveled that CCS2 induces AGS cells apoptosis through the Caspase-dependent endogenous mitochondrial apoptosis pathway.

In summary, CCS2 significantly and concentration-dependently inhibits the proliferation of AGS cells. CCS2 exerts an inhibitory effect on AGS cell growth, probably by inducing AGS cell apoptosis through the endogenous mitochondrial apoptotic pathway. With the developing understanding of the etiology of malignant tumors, traditional drugs therapy has undergone improvement and innovation. Various drug delivery systems use nanomaterials as carriers to provide new therapeutic strategies for tumor treatment (Makvandi et al., 2021; Ramezani Farani et al., 2022). The targeted implantation of *L. plantarum* YT013 into the tumor area using this technology will bring unlimited possibilities to overcome gastric cancer.

## Data Availability

The original contributions presented in the study are included in the article/Supplementary material, further inquiries can be directed to the corresponding author.
